# Patient with Wolff-Parkinson-White syndrome with intermittent pre-excitation under subarachnoid block for urological surgery

**DOI:** 10.4103/0019-5049.79899

**Published:** 2011

**Authors:** Rakesh Garg, Renu Sinha, PK Nishad

**Affiliations:** Department of Anaesthesiology and Intensive Care, All India Institute of Medical Sciences, Ansari Nagar, New Delhi, India

**Keywords:** Anaesthesia, intermittent pre-excitation, subarachnoid block, WPW syndrome

## Abstract

Wolff-Parkinson-White (WPW) syndrome is one of the pre-excitation syndromes in which activation of an accessory atrioventricular (AV) conduction pathway leads to bypass the AV node and cause earlier ventricular activation than the normal pathway. We report a patient with intermittent WPW syndrome who repeatedly manifested pre-excitation after subarachnoid block.

## INTRODUCTION

Wolff-Parkinson-White syndrome (WPW) is one of the pre-excitation syndromes in which activation of an accessory atrioventricular (AV) conduction pathway leads to bypass the AV node and cause earlier ventricular activation than the normal pathway.[1] The distinct electrocardiographic (ECG) characteristics of WPW syndrome are a short PR interval and a widened QRS interval with a delta wave. The manifestation of pre-excitation under anaesthesia can be challenging. We report a patient with intermittent WPW syndrome who repeatedly manifested pre-excitation after subarachnoid block.

## CASE REPORT

**Figure 1:**
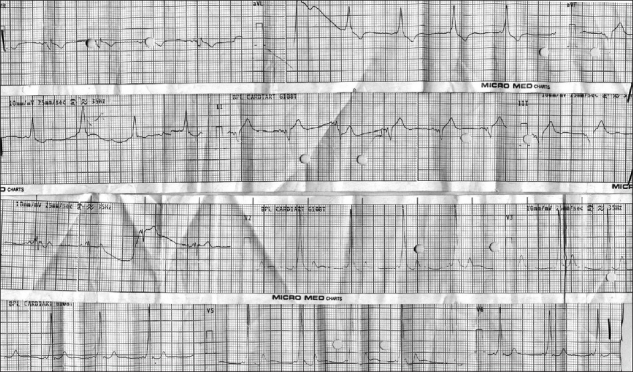
Preoperative ECG suggestive of Wolff-Parkinson-White syndrome

A 32-year-old male patient was admitted for urethroscopy and laser core through for stricture urethra. He did not give any history of palpitation, dyspnoea on exertion, syncope, dizziness or chest pain. On examination, patient had a pulse rate of 84 beats/min with occasional missed beats (4-5 per minute) and non-invasive blood pressure (NIBP) of 136/88 mmHg. On auscultation, chest was normal. ECG was suggestive of WPW syndrome (delta wave with broad QRS) with 4-5 ventricular ectopics [Figure 1]. Chest X-ray was normal. Echocardiography revealed normal valves, no regional wall motion abnormality with an ejection fraction of 55%. Holter evaluation revealed baseline sinus rhythm, WPW syndrome with right-sided pathway. Maximum heart rate (HR) achieved was 127/min with occasional ventricular premature beats.

**Figure 2:**
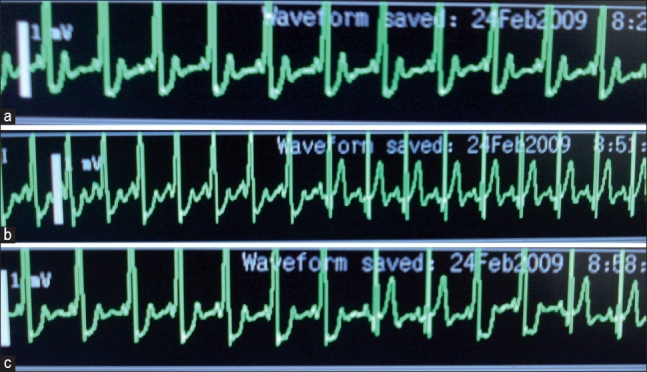
(a) Sinus rhythm followed by Wolff-Parkinson-White, (b) Sinus rhythm, (c) Wolff-Parkinson-White rhythm

Patient was premedicated with oral diazepam 10 mg the night before and on the morning of surgery. In the operation room, standard monitors (ECG, NIBP and pulse oximeter) were attached. The ECG showed normal sinus rhythm (NSR) with HR of 92-94 beats/min and occasional premature ventricular ectopics (4-5/minute). The 18-G intravenous was secured and 500 mL of ringer lactate was administered. The subarachnoid block was administered with 7 mg hyperbaric bupivacaine (0.5%) and 20 mcg fentanyl (total volume 1.8 mL) using 25-G pencil point spinal needle. The maximum sensory level achieved was T8. Supplemental oxygen was provided via face mask and midazolam (1 mg) was administered intravenously. Lithotomy position was made for the surgical procedure. After 30 minutes of surgery, ECG rhythm showed few slurred QRS rhythms with delta wave for 2 minutes with intermittent NSR followed by continuous rhythm suggestive of WPW syndrome [Figure 2a]. At this time HR was 72 beats/min and NIBP was 132/82 mm Hg. Oxygen saturation (SpO_2_) was maintained (98%). After 75 minutes, patient complained of slight discomfort in the back (probably because of the position) with increase in HR to 102 beats/minutes and NIBP of 130/80 mmHg. At this time, spinal block level was T8. The ECG rhythm changed to NSR for few seconds [Figure 2b] and again reverted back to WPW rhythm with HR 80-90 beats/min [Figure 2c] which remained till the end of surgery. During this period the NIBP ranged from 122 to 138/70-84 mmHg. The surgery lasted for 120 minutes. Two minutes before the end of procedure, the patient started feeling discomfort due to bladder expansion, which did not required further supplementation. After the surgery patient was shifted to post-anaesthesia care unit where 12 lead ECG revealed WPW syndrome rhythm with stable haemodynamics. Repeat ECG (12 hours and 48 hours, postoperatively) revealed NSR. Patient had an uneventful recovery.

## DISCUSSION

The incidence of pre-excitation syndrome varies from 0.1 to 3 per 1000 in healthy subjects.[1] The prevalence is higher in man and decreases with age due to loss of pre-excitation. Such patients usually have normal hearts but may be associated with various congenital and acquired defects (Epstein anomaly, mitral valve prolapse and cardiomyopathy).[1] In case of anterograde conduction via an accessory pathway, two parallel routes of conduction are possible, one subject to physiological delay over the AV node and the other passing directly without delay from atrium to ventricle. This sometimes may give an alternate conduction between the two pathways as had happened in our case.

The concerns in perioperative management of such cases include thorough evaluation of electrophysiological status, possibility of paroxysmal supraventricular tachyarrhythmia or atrial tachyarythmias or both and differentiation of ECG findings from myocardial infarction in the perioperative period.[2,3] The aim of the anaesthetic management should be the avoidance of tachyarrhythmias and sympathetic stimulation.

Although our patient was clinically asymptomatic but cardiac examination revealed presence of missed beats and ECG revealed WPW syndrome. An electrophysiological study should be performed in order to characterize and localize the accessory pathway. If the pathway is capable of conducting rapidly anterogradely, an immediate radiofrequency ablation is required prior to scheduled surgery due to risk of sudden death. Vigilance for patients with risk of myocardial infarction in perioperative period is needed as the anomalous complexes can mask or mimic myocardial infarction.[4] At times, WPW syndrome may not be detected at the preanaesthetic evaluation as a case of concealed WPW syndrome which was detected after spinal anaesthesia has been reported.[5]

For general anaesthesia, the anaesthetic plan should aim to reduce sympathetic outflow during periods of stress, such as induction and emergence.[4] Anaesthetic drugs tend to change the physiology of the AV conduction hence affect haemodynamics of the patient. Although regional anaesthesia avoids administration of multiple drugs and noxious stimuli of laryngoscopy, high block may lead to complications like blockade of cardiac sympathetic fibres and suppresses the normal AV conduction. Further, relative excitement of parasympathetic nerve due to high subarachnoid block facilitates conduction through accessory pathway. Hence the dosage of the drug for subarachnoid block should be administered very cautiously to avoid blocking of cardioaccelerator fibres. The impact of drugs for subarachnoid block and change in rhythm in WPW syndrome has not been mentioned in the literature. The autonomic nervous system is well-known to influence not only sinus node automaticity but also the refractoriness of the atrium and accessory pathway.[6] If sympathetic activity predominates, it will increase impulse generation at the sinus node, leading to the enhancement of conduction along both the AV node and antegrade conduction along the accessory pathway, which could result in the appearance of the delta waves and tachycardia.[6,7] In contrast in our case the faster HR reverted WPW rhythm to NSR intraoperatively. The possible reason for this change in rhythm could not be located in the literature.

Our patient had intermittent WPW syndrome based on detection of intermittent pre-excitation or intermittent loss of the delta wave during intraoperative ECG monitoring. During the perioperative period if some arrhythmias like an acute episode of reciprocating narrow QRS tachycardia occurs, then vagal maneuvers are tried initially if not successful then adenosine followed by intravenous verapamil or diltiazem are the other alternatives.[1] An external cardioverter-defibrillation is done in case of unstable tachycardia. In cases with wide QRS complex tachycardia, administration of procainamide or amiodarone should be considered. If atrial fibrillation is suspected from an anomalous QRS complex and grossly irregular R-R interval is present, drugs that prolong refractoriness in the accessory pathway (procainamide, propranolol) must be used. In all cases of manifest pre-excitation, administration of digoxin or verapamil is contraindicated due to risk of accelerating antegrade conduction via the accessory pathway. Moreover, preparation to perform electrical cardioversion in case of haemodynamic compromise or ventricular fibrillation also should be made.[8] Refractory arrhythmias under general anaesthesia has been managed with phenylephrine by acting directly to stimulate the arterial baroreceptors and hence vagal output.[9]

To conclude, choice of anaesthetic technique should be based on patient clinical condition and surgical requirement. In case of central neuraxial blockade, block level should be cautiously monitored and avoid higher level of blocks.
